# Evaluation of relationship between *TMPRSS2 p.(Val197Met)* variant and COVID-19 susceptibility and severity

**DOI:** 10.1186/s12879-024-08987-w

**Published:** 2024-01-22

**Authors:** Nora Ahmed Saleh Bashar, Nadida Mohammed Abdel-Hamid Gohar, Ahmed A. Tantawy, Mai Hamed Mohamed Kamel

**Affiliations:** 1 Department of Clinical and Chemical Pathology, Faculty of Medicine, Cairo University, Cairo, Egypt; 2 Department of Pulmonary Medicine, Faculty of Medicine, Cairo University, Cairo, Egypt

**Keywords:** Coronavirus disease 2019 (COVID-19), *TMPRSS2 gene variant*

## Abstract

**Background:**

The World Health Organization (WHO) declared Coronavirus Disease 2019 (COVID-19) a global pandemic on March 11, 2020. Severe Acute Respiratory Syndrome Coronavirus 2 (SARS-CoV-2) infection has killed millions of people and had a terrible effect on society. The transmembrane protease serine 2 *(TMPRSS2)* enzyme is essential in the initial phases of the interplay between the SARSCoV-2 and the host cells by assisting viral entrance.

**Methods:**

This observational case–control study involved 150 participants, 100 adult patients with COVID-19, 50 of whom appeared healthy and had no history of or symptoms of COVID-19 infection when the study was conducted. Between January and April 2022, patients were taken as inpatients in isolation units or through recruitment from the COVID-19 clinic at Kasr Al-Ainy Cairo University Hospitals. According to the National Institutes of Health guidelines (2021), they were categorised into three categories: mild, moderate, and severe. *TMPRSS2 p.(Val197Met)* variant genotyping was evaluated using TaqMan Real-Time PCR.

**Results:**

The study showed a substantial difference between the mild and severe COVID-19 patient groups regarding their *TMPRSS2 (p.Val197Met)* genotypes (*P* value = 0.046). The C allele was significantly more prevalent in the mild, moderate and severe COVID-19 patient categories (77.8%, 89.7% and 91.7%, respectively) and the control group (80%). Meanwhile, the T allele was more prevalent in the mild (22.2%) and control (20%) groups. There was a statistically significant difference in allelic distribution between the mild and severe groups (*P* value = 0.034).

**Conclusion:**

The study showed a connection between the *TMPRSS2* gene variant *p.(Val197Met)* and the degree of illness. We concluded that the T(mutant) allele was protective against severe COVID-19 because it was linked to lesser disease severity.

## Introduction

The Coronavirus disease 2019 (COVID-19) outbreak has become a significant issue for global public health. COVID-19 is caused by SARS-CoV-2 infection [1].

The transmembrane protease serine 2 *(TMPRSS2)* enzyme facilitates viral entry and is essential for SARS-CoV-2's initial interaction with host cells [2].

Given the broad spectrum of clinical symptoms associated with this illness, which range from asymptomatic to severe and life-threatening ones, genetic variations between patients may partially account for the diversity in disease symptoms. Most of these linked loci involve molecular activities such as viral entrance into host cells, immunological responses and inflammatory reactions [3].

A crucial human gene in SARS-CoV-2 infection is the *TMPRSS2* gene. It is found on chromosome 21 (21q22.3) and has a 492 amino acid reading frame and 15 exons [4].

Numerous *TMPRSS2* gene variants, particularly COVID-19, have been shown to impact the severity of viral infections. Methionine is substituted for valine at position 197 of the protein due to a missense mutation (C to T) at position 589 of the *TMPRSS2* gene. This variant is known as *p.Val197Met* *(TMPRSS2:c.589C* > *T)* [5].

Due to its location in the gene's coding region, the *TMPRSS2* variation may change how the encoded protein behaves. Several bioinformatics applications predicts that this mutation can reduce enzyme stability and activity, lessening SARS-CoV-2 activation and penetration into host cells. As a result, it has been hypothesized that this variant will guard against SARS-CoV-2 infection [6, 7].

The connection of the *TMPRSS2* gene variation with COVID-19 severity was investigated in several pieces of research, although the findings needed to be more consistent. More research is necessary to fully comprehend the intricate mechanisms underlying the relationship between *TMPRSS2* and SARS-CoV-2 and the significance of the *p.Val197Met* variant in determining disease severity [8].

## Material and methods

### Sample size

As considered the primary outcome, sample size calculation was done using the comparison of the prevalence of TMPRSS2 gene rs12329760 (p.Val197Met) polymorphism between COVID-19 patients and matched healthy controls. The calculation was done based on comparing 2 proportions from independent samples in a prospective study using the Chi test; the α-error level was fixed at 0.05, the power was set at 80%, and the group (case: control) ratio was set at 1. The sample size was calculated using PS Power and Sample Size Calculations software, version 3.0.11 for MS Windows (William D. Dupont and Walton D., Vanderbilt University, Nashville, Tennessee, USA).

### Study population

This observational case–control study was performed on 150 subjects: 100 patients diagnosed with COVID-19 admitted to Cairo University isolation hospitals or visited the COVID-19 clinic and 50 healthy individuals with no history or symptoms of COVID-19 infection at the time of the study. The patients were subdivided into three groups: mild, moderate and severe, according to the National Institutes of Health (NIH guidelines) 2021 *(COVID-19 treatment guidelines panel. Coronavirus Disease 2019 treatment guidelines. National Institutes of Health)*.

#### Inclusion criteria

Mild, moderate and severe COVID-19 patients diagnosed by the characteristic clinical picture, laboratory and radiological findings and confirmed by PCR test of their nasopharyngeal samples.

#### Exclusion criteria

Patients with any lung disease were excluded from this study.

### Sample collection

A thorough medical history was gathered from every patient, paying particular attention to their age, sex, history of COVID-19 exposure, need for a hospital stay and presence of comorbidities. Patients were subjected to chest CT and some routine laboratory investigations (CBC, CRP, LDH, D-dimer, Ferritin). In addition, 3 mL of each subject's venous blood was collected, and the blood was then emptied into sterile ethylene diamine-tetra-acetate (EDTA) tubes.

### Nucleic acid extraction

Genomic DNA was extracted from EDTA-anticoagulated blood and peripheral blood leucocytes using *EasyPure®* Blood Genomic DNA Kit * using spin columns. Extracted DNA samples were then kept at -20 °C until the time of the assay.

Amplification of extracted DNA and analysis of TMPRSS2 p.Val197Met gene variant (rs12329760) by Real-Time PCR technique using TaqMan single nucleotide variant genotyping assay:

Real-Time PCR allelic discrimination assay was designed using Taq-Man SNP Genotyping Assays (Applied Biosystems) and performed on the Step One *TM* Real-Time PCR System (Applied Biosystems). PCR with sequence-specific primers defined the *TMPRSS2* p.Val197Met gene variant.

### Statistical methods

Data entry, coding and analysis were done using IBM SPSS software suite version 23.0. Numbers and percentages were used to describe qualitative data. The distribution's normality was evaluated using the Kolmogorov–Smirnov test. The description of quantitative data used range (minimum and maximum), mean and standard deviation. The significance of the results was assessed at the 5% significance level. A chi-square test was performed to compare categorical variables between several groups. Fisher's exact test was employed to correct for chi-square when more than 25% of the cells had anticipated counts lower than 5. Student t-tests were employed to compare two research groups using normally distributed quantitative data. Mann–Whitney test was used to compare two groups under study when there were abnormally distributed quantitative data. ANOVA was utilized to compare data from more than two study groups when quantitative factors were involved.

## Results

This case–control study was carried out on one hundred patients with COVID-19 with a mean age of 48.56 ± 17.29 and fifty asymptomatic, apparently healthy individuals with a mean age of 43.46 ± 11.697. In the patient group, 55% were males, while in the control group, 44% were males. Comorbidities were substantially more prevalent in both the moderate and severe groups of cases compared to the mild group of COVID-19 patients according to clinical data and illness severity with percentages of 76.5% and 86.7%, respectively (*p*-value < 0.001). The inflammatory markers CRP, Ferritin and LDH were considerably higher in the moderate and severe cases than in the mild group (*p*-value = 0.019, 0.039 and < 0.001, respectively). Regarding the D-dimer level, there were no statistically significant variations between the three groups in Table 1.

**Table 1:** Clinical and demographic study data

**Variable**	**COVID-19 patients** **(*n* = 100)**			**Control group** **(*n* = 50)**	***P*-value**
	**Mild COVID-19 patients** **(*n* = 36**	**Moderate COVID-19 patients** **(*n* = 34)**	**Severe COVID-19 patients** **(*n* = 30)**		
**Age (years)**					
**Mean ± SD**	48.56 ± 17.29			43.46 ± 11.697	0.068
**Sex**					
**Males**	55 (55%)			22 (44%)	0.228
**Females**	45 (45%)			28 (56%)	
**Presence of comorbidity**					
Present	10 (27.8%)	26 (76.5%)	26 (86.7%)		< 0.001*
Absent	26 (72.2%)	8 (23.5%)	4 (13.3%)		
**CRP**					
**Mean ± SD**	43.43 ± 49.69	84.65 ± 63.77	82.49 ± 73.92		0.011*
Sig. between groups	p1 = 0.019*, p2 = 0.035*, p3 = 0.990				
**D-Dimer**					
**Mean ± SD**	1.08 ± 1.40	2.80 ± 4.81	1.80 ± 4.33		0.165
**LDH**					
**Mean ± SD**	273.25 ± 117.32	346.88 ± 179.50	498.70 ± 171.91		< 0.001*
Sig. between groups	p1 = 0.129, p2 < 0.001*, p3 = 0.001*				
**Ferritin**					
**Mean ± SD**	287.96 ± 274.26	566.62 ± 485.43	825.07 ± 616.69		< 0.001*
Sig. between groups	p1 = 0.039*, p2 < 0.001*, p3 = 0.077				

p is the *p*-value used to compare the examined groups; p_1_ is the *P*-value used to compare between Mild and Moderate; p_2_ is the *P*-value used to compare between Mild and Severe; p_3_ is the *P*-value used to compare between Moderate and Severe

*Statistically significant at *p* ≤ 0.05

**Fig. 1:**
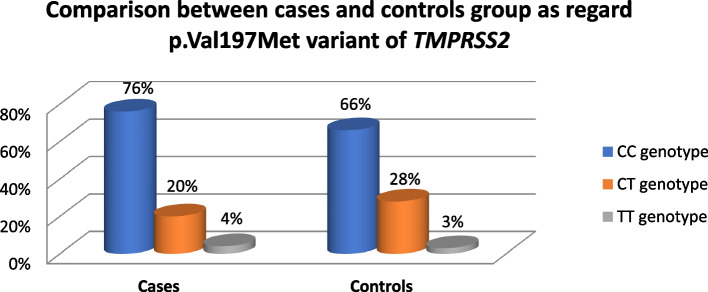
Genotype distribution of TMPRSS2 p.Val197Met variant between COVID-19 patients and control group

For 150 patient samples, the *TMPRSS2 p.Val197Met* genotypes were identified using the PCR technique. Each genotype and allele's frequency in the control and patient groups was computed. It was discovered that the CC genotype was more common in COVID-19 patients (76%) and the control group (66%) compared to the CT and TT genotypes. While the prevalence of the CT genotype was 20% in patients and 28% in the control group, the TT genotype was the least frequent in patients and controls, at a mere 4% and 3% respectively Fig. 1.

The differences between patients and the control group are not statistically different in genotypes, which were observed for the *p.Val197Met* variant (*P*-value = 0.449). Meanwhile, a distinction between mild and severe was found to be significant (*P*-value = 0.046) in Table 2. In addition, radiological findings were significantly more frequent in the CC genotype than in other genotypes in our study (*P*-value = 0.027). At the same time, there were no statistically significant differences regarding comorbidity or SpO2 in different genotypes Table 3.

**Table 2:** Comparison of the *TMPRSS2 p.Val197Met* genotypes in the three patient groups under study

Variable	Mild COVID-19 patients (*n* = 36)	Moderate COVID-19 patients (*n* = 34)	Severe COVID-19 patients (*n* = 30)	*P*-value
**Genotype**				
CC	22 (61.1%)	28 (82.4%)	26 (86.7%)	0.1009
CT	12 (33.3%)	5 (14.7%)	3 (10.0%)	
TT	2 (5.6%)	1 (2.9%)	1 (3.3%)	
**Sig. between groups**	p_1_ = 0.131, p_2_ = 0.046*, p_3_ = 0.855			

*Statistically significant at *p* ≤ 0.05

**Table 3:** Comparison among the three genotypes as regards clinical and radiological data

**Variable**	**CC** **(*n* = 76)**	**CT** **(*n* = 20)**	**TT** **(*n* = 4)**	***P*-value**
**Presence of Comorbidity**				
Present	49 (64.5%)	10 (50.0%)	3 (75%)	0.507
Absent	27 (35.5%)	10 (50.0%)	1(25%)	
**SpO2**				
≥ 94%	46 (60.5%)	17 (85.0%)	3 (75%)	0.096
< 94%	30 (39.5%)	3 (15.0%)	1(25%)	
**Radiological findings**				
Present	54 (71.1%)	8 (40.0%)	2 (50%)	0.027*
Absent	22 (28.9%)	12 (60.0%)	2 (50%)	

^*^Statistically significant at *p* ≤ 0.05

**Fig. 2:**
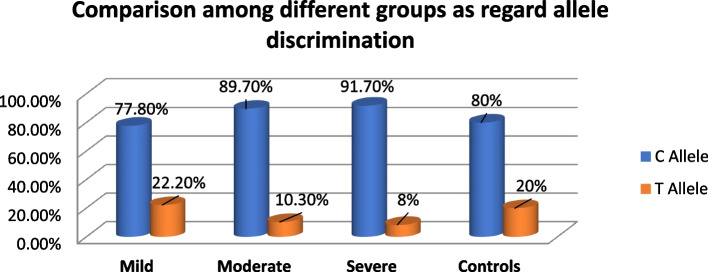
Allele frequencies of *TMPRSS2 p.(Val197Met)* variant between study groups of COVID-19 patients and control group

Compared to the T allele, the C allele was significantly more prevalent in the mild, moderate and severe COVID-19 patient categories (77.8%, 89.7% and 91.7%, respectively) and the control group (80%). When compared to the moderate (10.3%) and severe (8%) groups, the T allele was more prevalent in the mild (22.2%) and asymptomatic (20%) groups. There was a statistically significant variation in allelic distribution between the mild and severe groups (*P* value = 0.034) in Fig. 2.

## Discussion

The SARS-CoV-2 outbreak has resulted in a serious global crisis (COVID-19). During infection, a dense glycosylated spike protein (S protein) is required for SARS-CoV-2 to enter host cells. Furthermore, cell surface transmembrane protease serine 2 *(TMPRSS2)* cleaves and activates the S protein to promote membrane fusion and entrance [9, 10].

Among the 21 single nucleotide variants (SNVs) in the *TMPRSS2* gene, a computational study has revealed that the *p.Val197Met* variant affects this enzyme's structure by creating a pocket in the protein structure. This can lower enzyme activity and stability, which is anticipated to protect against SARS-CoV-2 infection by reducing SARS-CoV-2 activation and entrance into host cells [6–8].

Thus, we carried out case–control research to assess the contribution of the *TMPRSS2 p.(Val197Met)* gene variant to the susceptibility and severity of COVID-19.

In the current study, it was found that both the moderate and severe groups of cases were significantly older than the mild group. These findings were consistent with several studies conducted on COVID-19 patients, as the *Centers for Disease Control and Prevention, 2021* has reported that aging is one of the major risks for hospitalization, ICU admission and mortality due to COVID-19. They found that people aged 85 years and older had 350 times higher risk for mortality and 15 times more risk for ICU admission compared to younger age groups [11].

In our study, neither the sex of the patients nor the COVID-19 severity nor the patients' link to the control group were found to be significantly correlated by statistical analysis. This is consistent with another study, which discovered no appreciable difference in overall male and female *TMPRSS2* gene expression [12].

In the current study, medical comorbidities were significantly more frequent in moderate and severe cases than in the milder group. These findings were consistent with many reports that stated COVID-19 patients with diabetes, cardiovascular diseases, hypertension, chronic obstructive pulmonary disease (COPD), and malignancies are more prone to developing a life-threatening situation [13].

According to our results, CRP, LDH, and ferritin levels were considerably higher in the moderate and severe case groups than in the mild case groups. The three groups did not differ significantly in terms of D dimer levels.

*TMPRSS2 p.Val197Met* genotypes were not associated with age, sex, medical comorbidity, SpO2 and laboratory findings in our current study. Regarding the genotypic analysis in the current study, it was discovered that COVID-19 patients in the mild, moderate and severe groups had significantly greater frequencies of the C (wild) allele (77.8%, 89.7% and 91.7%, respectively) as well as the control group (80%) compared to the T allele in each of the groups.

However, the T (mutant) allele was found to be more frequent in the mild (22.2%) and control (20%) groups when compared to the moderate (10.3%) and severe (8%) groups. Therefore, there was statistically significant variation in the allelic distribution between the mild and severe groups (*P* value = 0.034).

David et al*. *2022 announced similar findings, showing that a decreased risk of severe COVID-19 was related to the T allele of the *p.Val197Met* variation (odd S ratio (OR) 0.87, 95% confidence interval (CI):0.79–0.97, *P* = 0.01). They found that the T allele reduces *TMPRSS2's* ability to catalyze, which reduces its capacity to facilitate SARS-CoV-2 spike-mediated entrance into host cells [14].

Moreover, our study demonstrated that COVID-19 patients (76%) had a greater prevalence of the CC genotype of the *TMPRSS2 p.Val197Met* variation than did the control group (66%) and was also higher in all patient groups compared to CT and TT. The COVID-19 patients' mild and severe groups differed significantly regarding their genotypes (*P* value = 0.046).

However, the findings of our current study were inconsistent with Yaghoobi et al*. *2023 who investigated the role of *TMPRSS2 p.Val197Met* genotype alleles among 251 Iranian COVID-19 patients who found that CC genotype was higher among the control group compared to the cases and higher among mild disease versus severe disease. However, none of those differences were statistically significant, with a *p*-value of 0.074. Additionally, the study discovered a statistically significant link between COVID-19 severity and the minor T allele [8].

A thorough investigation was also conducted in Iran to assess potential links between the *TMPRSS2 p.Val197Met* variation and vulnerability to SARS-CoV-2 acquisition and disease development. 1,285 COVID-19 patients were part of it. Although COVID-19-recovered patients had TT genotypes, patients with the *p.Val197Met* variation CC genotype showed noticeably increased COVID-19 infection mortality compared to those with other genotypes [15].

Together with our findings, these support the TT genotype's ability to protect against severe COVID-19.

An Egyptian study made by Abdelsattar et al*. *2022 demonstrated that there was an increase in the expression of genotype frequencies of ACE2 rs2285666 and *TMPRSS2* rs1232976 (TT), (CT + TT) and (T) alleles in the severe COVID-19 group compared to control and mild groups. The difference in the results may be related to the different *TMPRSS2* variants [16].

MIR et al. 2021 studied the strong association of angiotensin-converting enzyme-2 gene insertion/deletion polymorphism with susceptibility to SARS-CoV-2, hypertension, coronary artery disease and COVID-19 disease mortality. It was found that the ACE2-DD genotype was strongly associated with increased COVID-19 mortality. ACE2–CC and CT genotypes were also strongly associated with COVID-19 severity [17].

Ethnic differences might be proposed as one explanation for discrepancies in investigational findings. The patient’s genetic background likely affects the impact of T alleles on COVID-19 severity. These contradictory findings are caused by several *single nucleotide variant*s (SNVs) in the *TMPRSS2* gene, whose combined effects may complexly influence the course of the disease. Further research is required to resolve this issue.

## Conclusion

According to the study's findings, the *TMPRSS2* gene variant *p.Val197Met* and illness severity are related. We concluded that the T allele was linked to less severe COVID-19 and hence played a protective function. An additional study, including a bigger sample size and different populations, must confirm our findings.

## Data Availability

The datasets used and/or analyzed during the current study are available from the corresponding author upon reasonable request.

## Abbreviations

WHO: World Health Organization
COVID-19: Coronavirus Disease 2019
SARS-CoV-2: Severe Acute Respiratory Syndrome Coronavirus 2
CT: Computed tomography
TMPRSS2: Transmembrane protease serine 2
PCR: Polymerase chain reaction
NIH: National Institutes of Health
CBC: Complete blood count
CRP: C-reactive protein
LDH: Lactate dehydrogenase
EDTA: Ethylene diamine-tetra-acetate
DNA: Deoxyribonucleic acid
S protein: Spike protein
SNVs: Single nucleotide variants
COPD: Chronic obstructive pulmonary disease
SpO2: Saturation of Peripheral Oxygen
